# Inhibition of Jurkat T Cell Proliferation by Active Components of *Rumex japonicus* Roots Via Induced Mitochondrial Damage and Apoptosis Promotion

**DOI:** 10.4014/jmb.2007.07018

**Published:** 2020-10-20

**Authors:** Yinda Qiu, Aoding Li, Jina Lee, Jeong Eun Lee, Eun-Woo Lee, Namki Cho,, Hee Min Yoo

**Affiliations:** 1 College of Pharmacy, Chonnam National University, Gwangju 686, Republic of Korea; 2 Biometrology Group, Korea Research Institute of Standards and Science (KRISS), Daejeon 34113, Republic of Korea; 3 Metabolic Regulation Research Center, Korea Research Institute of Bioscience and Biotechnology (KRIBB), Daejeon 4141, Republic of Korea; 4 Department of Functional Genomics, KRIBB School of Bioscience, University of Science and Technology (UST), Daejeon 3113, Republic of Korea

**Keywords:** *Rumex japonicus* Houtt, apoptosis, reactive oxygen species (ROS), Jurkat cells

## Abstract

*Rumex japonicus* Houtt (RJH) is a valuable plant used in traditional medicine to treat several diseases, such as scabies and jaundice. In this study, Jurkat cell growth inhibitory extracts of *R. japonicus* roots were subjected to bioassay-guided fractionation, resulting in the isolation of three naphthalene derivatives (3-5) along with one anthraquinone (6) and two phenolic compounds (1 and 2). Among these compounds, 2-methoxystypandrone (5) exhibited potent anti-proliferative effects on Jurkat cells. Analysis by flow cytometry confirmed that 2-methoxystypandrone (5) could significantly reduce mitochondrial membrane potential and promote increased levels of mitochondrial reactive oxygen species (ROS), suggesting a strong mitochondrial depolarization effect. Real-time quantitative polymerase chain reaction (qPCR) analysis was also performed, and the results revealed that the accumulation of ROS was caused by reduced mRNA expression levels of heme oxygenase (HO-1), catalase (CAT), glutathione peroxidase (GPx), and superoxide dismutase (SOD). In addition, 2-methoxystypandrone (5) triggered strong apoptosis that was mediated by the arrest of the G0/G1 phase of the cell cycle. Furthermore, 2-methoxystypandrone (5) downregulated p-IκB-α, p-NF-κB p65, Bcl_2_, and Bcl-xl and upregulated BAX proteins. Taken together, these findings revealed that 2-methoxystypandrone (5) isolated from RJH could potentially serve as an early lead compound for leukemia treatment involving intracellular signaling by increasing mitochondrial ROS and exerting anti-proliferative effects.

## Introduction

Leukemia is a group of life-threatening malignant disorders of the bone marrow and blood that affects people of all ages from newborns to the elderly [1]. A key method of treating leukemia is chemotherapy; however, chemotherapeutic strategies remain limited by two primary issues: toxicity and the lack of efficacy [3, 5]. In recent years, research efforts have identified potent anti-cancer agents in several natural products and their analogs, which have garnered significant interest due to the limited number of associated side effects [6]. An important pathway being studied is apoptosis, which regulates homeostasis and morphogenesis by eliminating redundancy, which is closely associated with cancer in addition to other diseases [7].

Mitochondria are organelles enclosed by double membranes that serve to produce essential energy for cell metabolism. Most notably, mitochondria have been suggested to be at the center of the apoptotic pathway [8]. Mitochondrial membrane potential (ΔΨm) is used as an indicator of mitochondrial dysfunction, which can induce endogenous and exogenous ROS to alter mitochondrial membrane permeability as a cell undergoes cell death [9-12]. Therefore, many researchers see the potential of mitochondria as a target for cancer treatment. In our screening process using Jurkat cells, the methanolic extract of the roots of RJH was found to have significant anti-proliferative properties.

RJH is a perennial plant found in East Asia that belongs to the Polygonaceae family [13]. In traditional medicine, RJH roots are often used to treat jaundice, constipation, and other symptoms [14, 15]. Modern research has found that RJH roots, which contain large amounts of quinone, xanthone, and flavonoid structures, have strong antibacterial and antifungal properties [16, 17]. In our search for anti-leukemia agents against Jurkat cells, we found a strong potential candidate in 2-methoxystypandrone with a 1,4-dicarbonyl structure. Several reports have shown that 2-methoxystypandrone, which structurally belongs to the naphthalene group, exhibits antimicrobial activity in gram-positive bacteria and inhibitory growth activity in human cancer cells. However, researchers have yet to determine the mechanism of action of 2-methoxystypandrone in leukemia cell lines.

In the present study, we explore the ways in which 2-methoxystypandrone affects cell proliferation and oxidative stress induction in Jurkat cell lines. We confirmed that 2-methoxystypandrone displays potent mitochondrial depolarization by increasing ROS generation and decreasing the mRNA expression levels of antioxidant enzymes. Furthermore, we studied the mechanisms behind the apoptotic effects of 2-methoxystypandrone by evaluating the protein expression levels of Bcl_2_, Bcl-xl, BAX, p-IκB-α, and p-NF-κB p65.

## Materials and Methods

### Plant Materials

Roots of RJH were collected by H. S. Park (from the Medicinal Plant Garden, Boseong, Jeollanam-do, Korea), and a voucher specimen was deposited in the Herbarium of the Medicinal Plant Garden, College of Pharmacy, Chonnam National University, Korea.

### Extraction and Isolation

Roots of dried RJH (1.9 kg) were placed in 100% methanol and ultrasonicated three times. After evaporation of the solvent under a vacuum at 40°C, the total extract (325.2 g) was dissolved in water and fractioned successively and consecutively with ethyl acetate (EtOAc; 73.2 g) and n-butanol (86.2 g). The EtOAc fraction exhibited more potent levels of toxicity on Jurkat leukemia cells than the other fractions, and thus it was chromatographed on a silica gel column using a gradient of increasing polarity. A CH_2_Cl_2_ and MeOH mixture (CH_2_Cl_2_-MeOH 100:1 to 1:100, v/v) was used as a solvent to yield five fractions (E1-5). The five fractions were subjected to reverse phase (RP) C_18_ medium-pressure liquid chromatography (MPLC) with MeOH from 10% to 100%. The subfractions were then purified by high-performance liquid chromatography (HPLC) and eluted with acetonitrile (**A**) and H2O (**B**) to obtain compounds 1–6. E1 (5.44 g) was subjected to MPLC, and subfractions M1–6 were obtained. Compound 4 (4.6 mg) was isolated from subfraction M1 via HPLC with equilibration at 60% A. Subfraction M2 was further purified via HPLC using a 250 × 10 mm Phenomenex Luna C_18_ column with 50% A to generate compound 2 (4.1 mg). From subfraction M3, compound 3 (24.2 mg) was obtained via HPLC with a linear gradient profiled from 45% to 90% A. Compound 5 (26.3 mg) was isolated from subfraction M4 via HPLC using the same C_18_ HPLC column with equilibration at 60% A. RP C_18_ MPLC was performed on E2 (2.60 g), yielding subfractions M7-11. Subfraction M10 was further purified using a linear gradient profile from 20% to 90% A to produce compound 6 (12.3 mg). Subfraction M13 was subjected to HPLC with equilibration at 15% A to obtain Compound 1 (5.4 mg).

### Cell Culture

Jurkat cells were cultured in a 5% CO_2_ incubator with a humidified atmosphere and a temperature of 37°C for 2 to 3 days. The cells were supported using Advanced Roswell Park Memorial Institute (RPMI) 1640 medium supplemented with 2% fetal bovine serum and 1% penicillin‐streptomycin. The cells were subsequently subcultured at a ratio of 1:4 upon reaching a cell density of 70-80%.

### Cell Viability Assay

For the toxicity screening assays, the Jurkat cells were seeded in 96-well plates at a density of 1 × 10^5^ cells/well. A 10% (v/v) solution of 20 μl CellTiter One Solution Reagent was added to each well. The samples were then incubated for 2 h under a temperature of 37°C. The antiproliferative effects were measured using the MTS (3-(4,5-dimethylthiazol-2-yl)-5-(3-carboxymethoxyphenyl)-2-(4-sulfophenyl)-2H-tetrazolium, inner salt) assay, as described in our previous study [2]. The effects on cell growth were expressed as a percentage of the control.

### Microscopic Analysis

A phase-contrast microscope (Olympus, Japan) was used to examine any morphological changes in the Jurkat cells. Photomicrographs of the cells were taken at 24 h and 48 h after treatment with 2-methoxystypandrone and an analysis was performed to determine changes in the number of cells [18].

### Tetramethylrhodamine Methyl Ester Perchlorate (TMRM) Assay

To detect the mitochondrial membrane potential, the Jurkat cells were incubated with a fluorescent indicator, specifically 100 nM TMRM (Thermo Fisher Scientific, USA). The cells were washed in phosphate-buffered saline (PBS) and resuspended in fluorescence-activated cell sorting (FACS) buffer (PBS supplemented with 1% fetal bovine serum). The analysis of the cells involved the use of a flow cytometer (BD FACSVerse; BD Biosciences, USA) and the FlowJo software (FlowJo LLC, USA) [19].

### Determination of Intracellular Levels of Reactive Oxygen Species (ROS)

The Jurkat cells were seeded with a density of 1 × 10^5^ cell/ml, treated with 2-methoxystypandrone (10 μM), and exposed to ascorbic acid (AA) for 24 h. The cells were then incubated with 5 μM MitoSOX Red Mitochondrial Superoxide Indicator (Thermo Fisher Scientific). A flow cytometer (BD FACSVerse; BD Biosciences) and the FlowJo software (FlowJo LLC) were used to measure and analyze intracellular fluorescence ROS levels [20].

### Cell Cycle Analysis

The Jurkat cells were seeded in 25 cm^2^ flasks and exposed to 2-methoxystypandrone (10 or 20 μM) for 48 h. The cells were then collected and fixed in ice-cold 70% ethanol at 4oC for 6 h. After 5 min of centrifugation at 1,500 ×*g*, a premixed reagent that included the nuclear DNA intercalating stains propidium iodide (PI) and RNAse A was used to stain the cells, which is required to conduct analyses with the Muse Cell Cycle Assay Kit (Luminex Corporation, USA). The percentage of cells in each cell cycle phase was determined with the FlowJo software (FlowJo LLC) [20].

### Apoptosis Assay

Apoptotic cell populations were determined via staining with Annexin V-APC and propidium iodide (PI) according to the manufacturer’s instructions. The Jurkat cells were seeded at a density of 1 × 10^5^ cell/ml and treated with 2-methoxystypandrone (10 or 20 μM) for 48 h. The cells were harvested, washed with ice-cold PBS, and incubated with an apoptosis detection kit (BioLegend, USA) for 30 min. Apoptotic cell populations were measured using a flow cytometer (BD FACSVerse; BD Biosciences) and analyzed using the FlowJo software (FlowJo LLC) [21].

### Protein Extraction and Western Blot

The Jurkat cells were seeded at a density of 1 × 10^5^ cell/ml in 25 cm^2^ flasks and treated with 2-methoxystypandrone (10 or 20 μM) for 48 h. The cells were subsequently harvested and lysed with RIPA lysis buffer (Thermo Fisher Scientific) containing 1× protease inhibitor and phosphatase inhibitor cocktails (Roche Molecular Biochemicals, Switzerland). Bio-Rad protein assay reagents (Bio-Rad, USA) were used to determine the protein content, and an equal amount of protein was separated by sodium dodecyl sulfate (SDS)-PAGE (NuPage, Bis-Tris Gel 4-12%; Thermo Fisher Scientific). The protein was transferred onto a polyvinylidene difluoride (PVDF) membrane and then immunoblotted with specific primary antibodies for Bcl_2_, Bcl-xl, BAX, p-IκB-α, p-NF-κB p65, and β-actin at 4oC overnight with gentle shaking. Bound antibodies were detected using Immobilon Western Chemiluminescent HRP Substrate (Millipore, USA). Images were taken with an ImageQuant LAS 4000 Mini (Fujifilm, Japan) [22].

### Real-Time Quantitative Polymerase Chain Reaction (RT-qPCR)

Total RNA was isolated from the Jurkat cells via 2-methoxystypandrone (10 or 20 μM) treatment using the RNeasy Mini Kit (Qiagen, Germany) according to the manufacturer’s instructions. The iScript cDNA Synthesis Kit (Bio-Rad) was used to reverse transcribe the total RNA. The resulting cDNA was used for real-time qPCR amplification with specific primers and iTaq Universal SYBR Green Supermix (Bio-Rad) according to the manufacturer’s instructions. The reaction was performed in triplicate using the StepOnePlus Real-Time PCR system (Thermo Fisher Scientific), and the resulting values were normalized to housekeeping genes (β-actin or glyceraldehyde 3-phosphate dehydrogenase (GAPDH)) [22].

### Live/Dead Assay

To analyze the Jurkat cell counts, both living and dead cells underwent fluorescent labeling using the Live/Dead Viability/Cytotoxicity Kit (Thermo Fisher Scientific). Jurkat cells were stained for 30 min in the dark with a calcein and ethidium homodimer (EthD-1) following the manufacturer’s instructions. A fluorescence microscope (Olympus) was used to obtain images.

### Statistical Analyses

Statistical analyses were performed using GraphPad Prism 5 (GraphPad Software, Inc., USA). The values are provided as means ± S.D. The data were further analyzed using the one-way Student’s t-test and p-values (* *p* < 0.05, ** *p* < 0.01, *** < 0.001) were considered statistically significant [2, 4].

## Results

### Structural Elucidation of Compounds 1–6

**Fig. 1:**
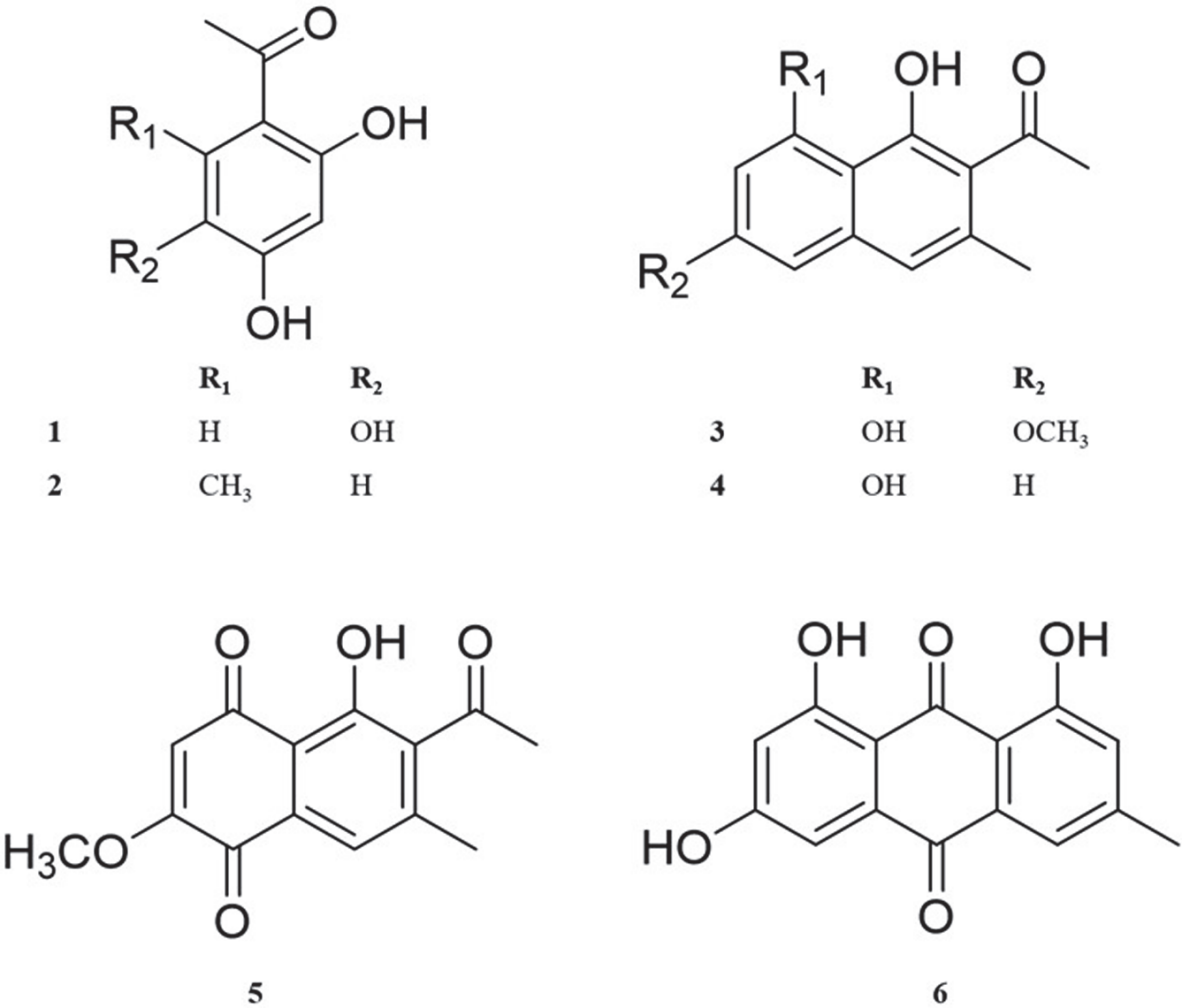
Chemical structures of compounds 1-6 isolated from the ethyl acetate (EtOAc) fraction of *Rumex japonicus* Houtt roots.

By subjecting the methanolic extract of RJH roots to bioassay-guided fractionation, we found that the EtOAc fraction exerted antiproliferative effects on the Jurkat cells. Further separation of the EtOAc fraction via activity-guided chromatographic methods yielded six known compounds with the following structures: 2,4,5-trihydroxyacetophenone (1), orcacetophenone (2), trachrysone (3), musizin (4), 2-methoxystypandrone (5), and emodin (6). The structures were determined based on comparisons with data in the literature (Fig. 1) [23-26].

### Compound-Induced Antiproliferative Effects

**Fig. 2:**
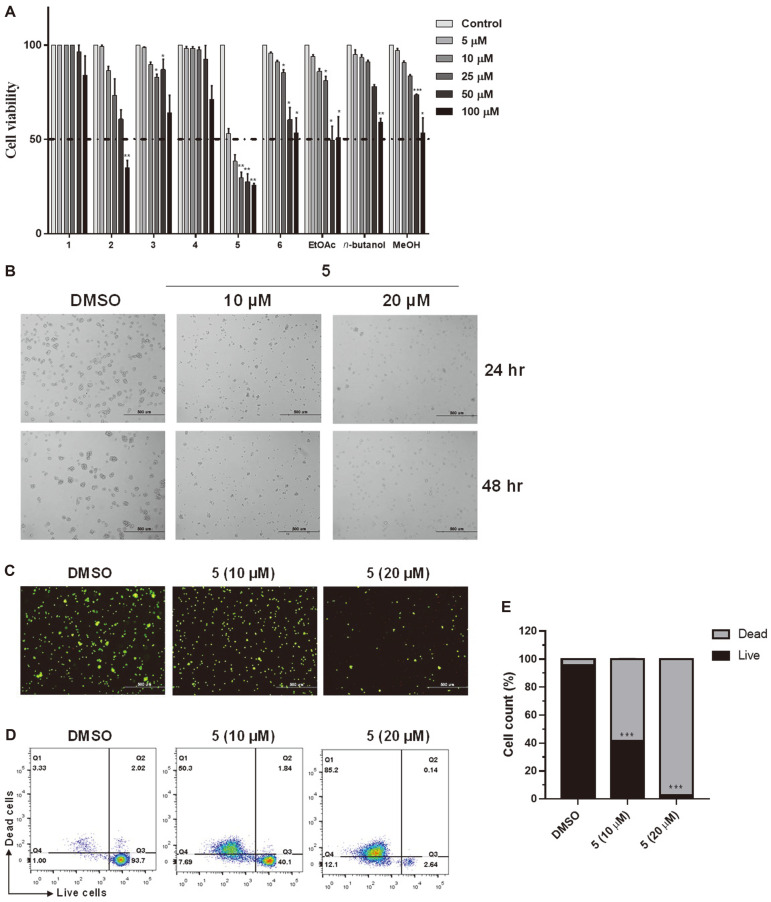
Antiproliferative effects of compounds 1–6 and fractions in Jurkat cells. The initial concentrations of EtOAc, n-Butanol, and MeOH are 100 µg/ ml. (**A**) Cell viability as assessed by the MTS assay 48 h post-treatment. (**B**) Antiproliferative effects of 2-methoxystypandrone (5) in Jurkat cells (*n* = 3,200 × magnification, scale bar: 500 μm). The bar chart of all data represents mean ± SEM (*n* = 3). * *p* < 0.05, ** *p* < 0.01, and *** *p* < 0.001 versus the control group. (**C**) Fluorescence images showing 48 h of 2-methoxystypandrone-treated and untreated Jurkat cells (green, live cells; red, dead cells). (**D**) Flow cytometry analysis of live and dead Jurkat cells after calcein and ethidium homodimer-1 (EthD-1) staining. (E) Quantified live and dead cells. The bar chart of all data represents mean ± SEM (n=3). *p < 0.05, **p < 0.01, and ***p < 0.001 versus the control group.

The cytotoxic potentials of the six compounds that were isolated from RJH roots were determined by the MTS assay. Among the compounds, 2-methoxystypandrone (5) exhibited the strongest antiproliferative activity at concentrations ranging from 25 μM (29.8 ± 4.1%) to 100 μM (26.3 ± 2.2%) (Fig. 2A). Cell viability was dramatically reduced even at lower concentrations such as 5 and 10 μM, suggesting that compound 5 could be a powerful anti-cancer agent for inhibiting leukemia cell proliferation (Fig. 2A). In addition, examinations under a microscope showed that morphological changes characteristic of apoptosis were induced in cells treated with 2-methoxystypandrone compared to cells treated with dimethyl sulfoxide (DMSO), as shown in Fig. 2B. It is noteworthy that the antiproliferative activity of 2-methoxystypandrone was superior to that of compounds 3 and 4 despite the structural similarities between the three compounds. The differences in the antiproliferative activity of these compounds may be explained by the existence of a double carbonyl group. Furthermore, upon comparing 2-methoxystypandrone to compounds 1 and 2, it was determined that a 1,4-naphthoquinone group is an important structural feature for antiproliferative activity. Based on these data, we chose 2-methoxystypandrone for further research.

The Live/Dead assay utilizes two fluorescent dyes, calcein (live-cell) and ethidium homodimer-1 (EthD-I, dead-cell). The live and dead cells were imaged by fluorescence microscopy (Fig. 2C). The 2-methoxystypandrone-treated Jurkat cells exhibited (in a dose-dependent manner) reduced levels of green fluorescence-labeled live cells and induced levels of red fluorescence-labeled dead cells within 48 h of exposure compared to the DMSO control (Figs. 2D and 2E). As such, it is clear that 2-methoxystypandrone markedly increased the population of apoptotic cells.

### Changes in Mitochondrial Membrane Potential

**Fig. 3:**
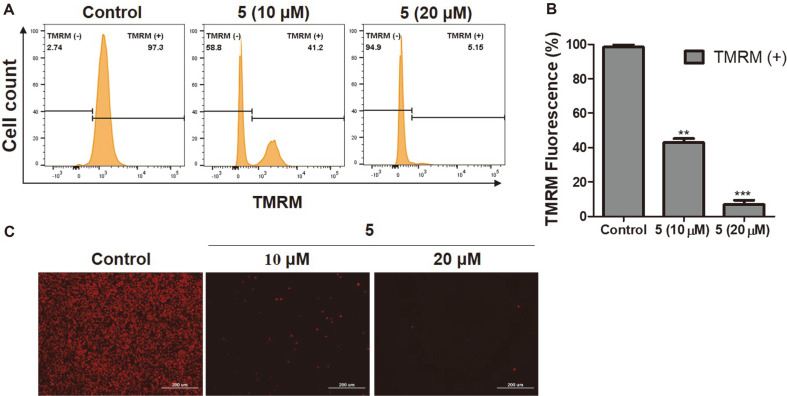
Changes in mitochondrial membrane potential in Jurkat cells. (**A**) Mitochondrial membrane potential in Jurkat cells loaded with 100 nM tetramethylrhodamine methyl ester perchlorate (TMRM) as analyzed by flow cytometry. (**B**) Quantification of TMRM-positive fluorescence for mitochondrial membrane potential. (**C**) Detection of mitochondrial membrane potential based on fluorescence intensity measurements via fluorescence microscopy. The bar chart of all data represents mean ± SEM (*n* = 3). **p < 0.01 and ***p < 0.001 versus the control group.

In cancer cell apoptosis studies, mitochondrial membrane potential (ΔΨm) is a key indicator to evaluate the function and state of mitochondria. To elucidate whether the cytotoxic effect of 2-methoxystypandrone is related to the mitochondrial membrane, we measured mitochondrial depolarization by probing TMRM fluorescence intensity through flow cytometry. According to the results, mitochondrial depolarization was significantly increased by the 2-methoxystypandrone treatment (Figs. 3A and 3B). Microscopic data also showed that, compared to the DMSO group, the Jurkat cells exhibited reduced TMRM-positive fluorescence (Fig. 3C). Thus, the findings suggest that 2-methoxystypandrone significantly downregulates mitochondrial membrane potential levels in a dose-dependent manner.

### ROS Changes in Jurkat Cells

**Fig. 4:**
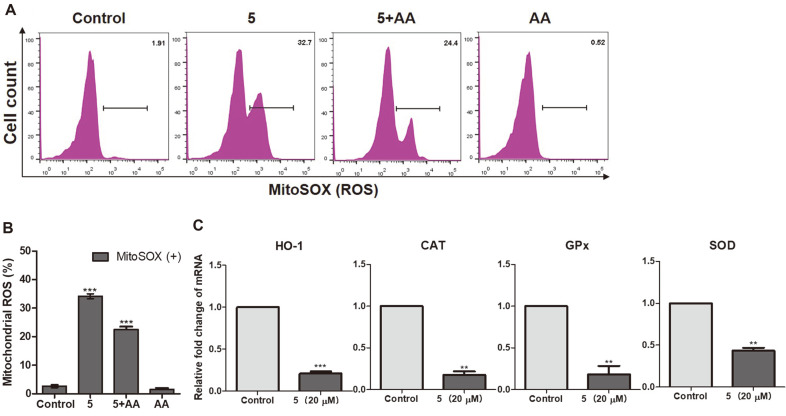
2-methoxystypandrone-induced mitochondrial reactive oxygen species (ROS) in Jurkat cells. (**A**) Mitochondrial ROS levels in 2-methoxystypandrone-treated Jurkat cells after 24 h as detected by flow cytometry with MitoSOX (5 μM). (**B**) Quantified mitochondrial ROS levels. (**C**) Real-time quantitative polymerase chain reaction (qPCR) analysis results of the mRNA levels of heme oxygenase (HO-1), catalase (CAT), glutathione peroxidase (GPx), and superoxide dismutase (SOD) in the control and treated Jurkat cell groups. The values indicate means ± SEM (*n* = 3, ***p < 0.001).

Mitochondrial membrane potential can induce ROS to change mitochondrial membrane permeability and promote cancer cell death. We previously demonstrated that 2-methoxystypandrone can interfere with mitochondrial depolarization. Next, we investigated how this interference affects the production of ROS. The results showed that ROS levels increased from 1.91% (control) to 32.7% (2-methoxystypandrone treatment). At the same time, ROS accumulation was reduced by AA, an ROS scavenger (Figs. 4A and 4B). The MitoSOX Red staining results agree with the mitochondrial potential data, indicating that 2-methoxystypandrone serves as an inducer of oxidative stress, which generates significant mitochondrial ROS. Heme oxygenase (HO-1), catalase (CAT), glutathione peroxidase (GPx), and superoxide dismutase (SOD) are several key enzymes that participate in mediating antioxidant effects. Real-time qPCR analysis revealed that the mRNA expression levels of the aforementioned antioxidant enzymes had significantly decreased (Fig. 4C). Therefore, these findings demonstrated that 2-methoxystypandrone was used as an oxidant to downregulate antioxidants, which subsequently led to the accumulation of ROS.

### Cell Cycle Distribution of 2-Methoxystypandrone-Induced Jurkat Cells

**Fig. 5:**
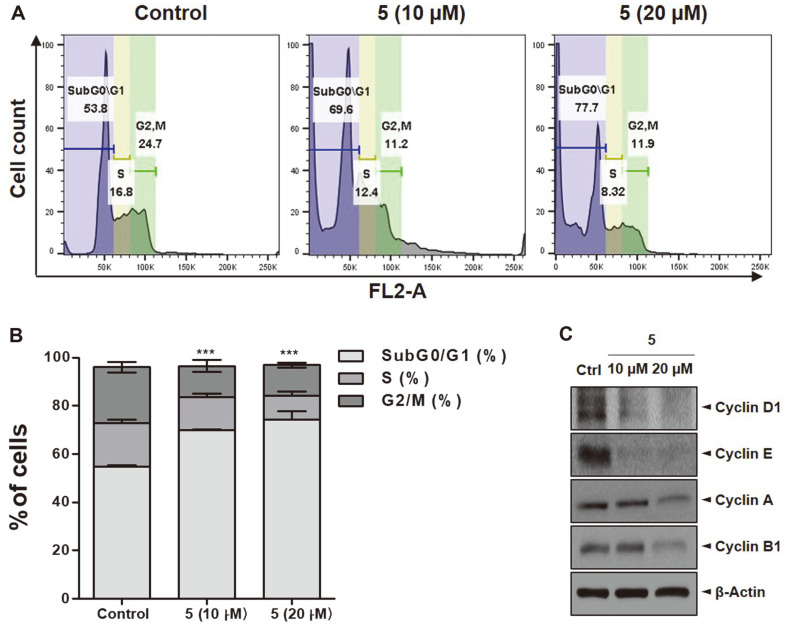
Changes in cell cycle progression induced by 2-methoxystypandrone in Jurkat cells. (**A**) Percentage of cells in the sub-G0/G1, S, or G2/M phases after treatment with 2-methoxystypandrone as analyzed by flow cytometry. (**B**) Quantification of cells in the G0/G1, S, or G2/M phases. The bar chart of all data represents mean ± SEM (*n* = 3). ***p < 0.001 versus the control group. (**C**) Cyclin D1, E, A, and B1 protein levels as determined by western blotting.

We further investigated how 2-methoxystypandrone treatment affected the cell cycle of the Jurkat cells. The results showed that the sub-G0/G1 phase increased in a dose-dependent manner from 53.8% in the DMSO group to 69.6% (10 μM) and 77.7% (20 μM) in the experimental groups (Fig. 5A). The increase in the G0/G1 subpopulation was accompanied by a significant reduction in cell subpopulations in the S and G2/M phases (Fig. 5B).

Cancer is said to be caused by uncontrolled proliferation resulting from the aberrant activity of cell cycle regulators [27]. The cell cycle has four distinct phases called G1, S, G2, and M, and the progression of this cycle is regulated by heterodimeric complexes formed by cyclin D, cyclin E, cyclin A, or cyclin B with several cyclin-dependent kinases (CDKs) [28]. To identify whether 2-methoxystypandrone-induced cell cycle arrest was due to changes in the protein levels of cell cycle regulators, we further examined the expression levels of cyclins. Relative to the control, 2-methoxystypandrone markedly decreased the expression levels of cyclin D1, E, A, and B (Fig. 5B). The results supported that 2-methoxystypandrone could induce cell cycle arrest at the S, and G2/M phases in Jurkat cells (Fig. 5C).

### Apoptosis Analysis of 2-Methoxystypandrone-Induced Jurkat Cells

**Fig. 6:**
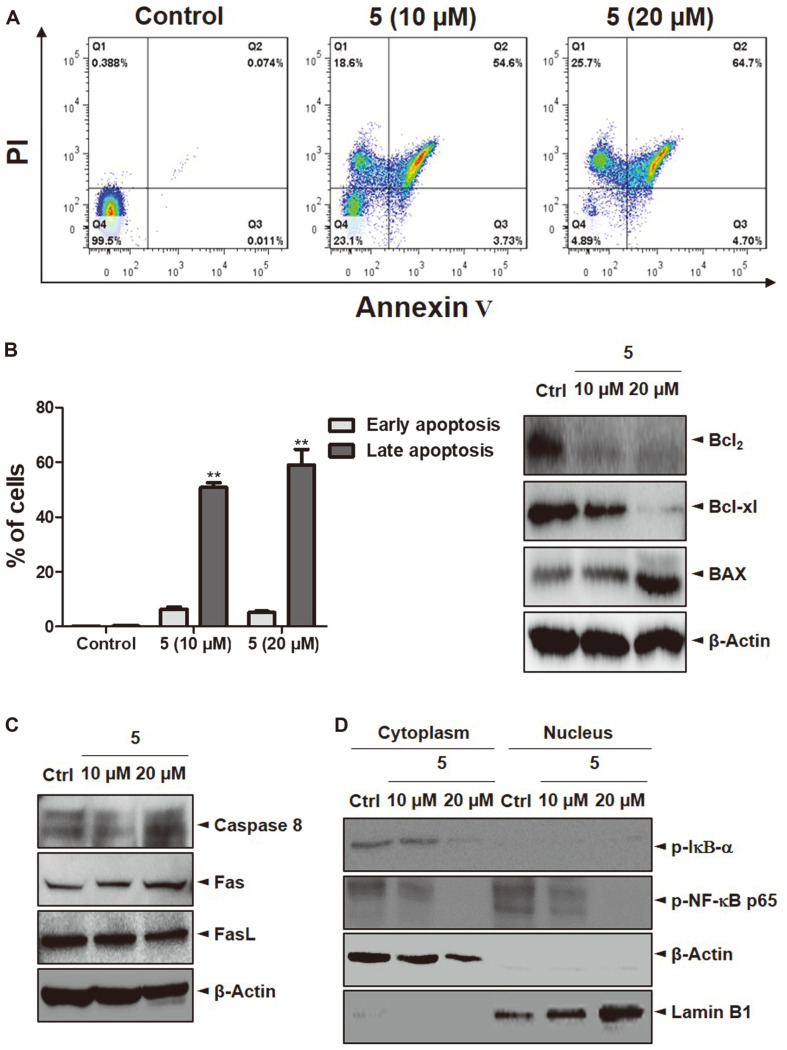
Apoptosis level changes in Jurkat cells induced by 2-methoxystypandrone. (**A**) Jurkat cells were either treated with 2-methoxystypandrone or untreated for 48 h, and apoptosis was subsequently evaluated by flow cytometry. (**B**) Quantification of early and late apoptotic cells. The bar chart of all data represents mean ± SEM (*n* = 3). **p < 0.01 versus the control group. (**C**) Protein expression levels of Bcl_2_, Bcl-xl, and BAX in the Jurkat cells as examined by western blot analysis. (**D**) Fas, FasL, and caspase-8 protein levels as determined by western blotting. (**E**) The cells were treated with 2-methoxystypandrone for 48 h before the cytoplasmic and nuclear cell fractions were analyzed for p-IκB-α and p-NF-κB p65 expression levels via western blotting.

Jurkat cells were used to evaluate apoptosis induced by 2-methoxystypandrone. Annexin V and PI dyes were used to double-stain the cells, which is necessary to determine the percentages of early and late apoptotic cells as well as viable cells. After 48 h, the percentage of late apoptotic cells had significantly increased whereas the percentage of live cells had decreased, confirming that 2-methoxystypandrone induced apoptosis (Figs. 6A and 6B). To determine the molecular mechanisms, apoptosis-related protein levels were analyzed via western blotting. According to the results, anti-apoptotic proteins such as Bcl_2_ and Bcl-xl saw significantly downregulated expression levels, whereas BAX, a pro-apoptotic protein, saw upregulated expression levels (Fig. 6C). The extrinsic pathway induces apoptosis through external stimulation: for example, Fas ligand (FasL). Initiator caspases such as caspase-8 can be activated by death receptors such as Fas through dimerization mediated by adaptor proteins. Upon activation, caspase-8 cleaves and activates the effector caspase-3, which leads to apoptosis [29, 30]. To elucidate whether the extrinsic signaling pathway of apoptosis is involved or not, a western blot analysis involving two concentrations (10 and 20 μM) of 2-methoxystypandrone was performed. The western blots showed that 2-methoxystypandrone did not change the protein levels of Fas, FasL, and caspase-8. These data show that the Fas/FasL-mediated pathway is not activated during 2-methoxystypandrone-induced apoptosis (Fig. 6D).

NF-κB p65 is a key factor that is extensively involved in cancer development and progression [31]. The NF-κB family is overexpressed in several cancer cells and is inactive in the cytoplasm due to the IkB inhibitory subunits [32]. During tumor promotion, apoptosis is avoided via an important step involving the activation of NF-κB. Therefore, the blockading of NF-κB is an interesting approach to suppressing cancer development [33]. For this reason, we determined whether 2-methoxystypandrone affected phosphorylated NF-κB p65 and phosphorylated IκBα. Compared to the control, phospho-NF-κB p65 levels were markedly decreased in both the cytoplasm and nucleus fraction, and phospho-IκBα expression levels were significantly decreased in the cytoplasm following treatment with 2-methoxystypandrone. This suggests that the inhibition of the NF-κB signaling pathway is involved in 2-methoxystypandrone-induced apoptosis in Jurkat cells. Therefore, a potential therapeutic application of 2-methoxystypandrone may be as an anticancer drug for the treatment of lymphoma (Fig. 6E).

## Discussion

In recent years, a wide array of bioactive plant components have received immense attention due to their potential anticancer effects [34]. New drugs could potentially be discovered by studying natural compounds; with their diverse structures, it may be possible to develop drugs capable of targeting abnormal molecular and biochemical signals that lead to cancer [35]. In this study, we purified six compounds from naturally growing RJH roots and showed that 2-methoxystypandrone (5) exhibited significant antiproliferative effects on Jurkat cells. In addition, 1,4-naphthoquinones such as 2-methoxystypandrone have demonstrated noteworthy cancer prevention potential [36]. The 1,4-naphthoquinones are redox-active compounds that are structurally related to naphthalene and contain a benzene moiety linearly fused with a fully conjugated cyclic diketone in which the carbonyl groups are arranged in *para* orientation [37]. Much of the biological activity of 1,4-naphthoquinones can be explained by acid-base properties in addition to that fact that they have two carbonyl groups that can accept one or two electrons to frame the corresponding radical anion or dianion species [38]. According to a previous study, 2-methoxystypandrone displayed superior antiproliferative activity on cervical cancer [39]. In our experimental research with Jurkat cells, 2-methoxystypandrone also exhibited more potent cytotoxic effects compared to compounds 3 and 4, which have a naphthol moiety.

The cell cycle is a complex process that is important when considering cell proliferation and the regulation of DNA damage repair. The cycle can be divided into the DNA synthesis phase (G0/G1), the DNA replication phase (S), and the cell division phase (G2/M) [40]. In our study, we confirmed that 2-methoxystypandrone inhibited Jurkat cell proliferation by blocking the G0/G1 phase. Compared to the DMSO group, cells treated with 2-methoxystypandrone experienced arrest at the G0/G1 phase in a dose-dependent manner (Figs. 5A and 5B). According to certain studies, the G0/G1 phase is a critical period for DNA synthesis. If blockage occurs during this period, impacted cells cannot enter the DNA replication phase, which in turn affects normal cell division [41]. It appears that 2-methoxystypandrone exerts antiproliferative effects by interfering with DNA synthesis. Furthermore, cell cycle regulators induced apoptosis and inhibited proliferation. In Annexin V/PI double staining, the percentage of late apoptotic cells was greater in the 2-methoxystypandrone treatment group compared to the DMSO group, a trend that was dose-dependent (Figs. 6A and 6B). Apoptosis, the most important form of cell death, is driven by both the mitochondrial and cell death receptor pathways. At the center of the apoptotic pathway are mitochondria, which provide a number of key factors. For example, several key members of the Bcl_2_ family rely on mitochondria to function. Proteins of this family, which control the intrinsic apoptosis pathway and serve as important regulators of cell apoptosis, can be divided into pro-apoptotic and anti-apoptotic proteins [42]. Research indicates that Bcl_2_ was discovered in acute lymphocytic leukemia and was later proven to protect cells from programmed cell death. Bcl_2_ and Bcl-xl exert some anti-apoptotic effects by regulating mitochondrial homeostasis [43]. On the other hand, the pro-apoptotic protein BAX can alter the permeability of mitochondrial membranes and promote apoptosis. In this study, we demonstrated that the expression levels of Bcl_2_ and Bcl-xl, key anti-apoptotic proteins of the Bcl_2_ family, had significantly decreased whereas that of BAX had increased (Fig. 6C), which may explain the induction of apoptosis following 2-methoxystypandrone treatment. Subsequently, we explored the anti-proliferative mechanism of 2-methoxystypandrone. NF-κB activation has been reported in many human cancers and plays a key role in oncogenesis and tumor growth [44]. When the NF-κB pathway is stimulated, the IKK complex is activated via signal transduction, which promotes the expression of IκB-α and ultimately allows the p65/p50 heterodimer to be transported to the nucleus, regulating the downstream NF-κB signaling pathway [45]. Our results also demonstrated that 2-methoxystypandrone induced apoptosis by downregulating p-NF-κB p65 and p-IκB-α in Jurkat cells (Fig. 6D).

Several recent studies have indicated that certain mitochondrial features are different in cancer cells compared to those in normal cells, including mitochondrial membrane potential and ROS levels [46]. Therefore, these features could serve as important indicators of mitochondrial dysfunction in tumor cells. Mitochondrial membrane potential is related to apoptosis as its dissipation is a critical event in the apoptosis process. On the other hand, ROS can affect mitochondrial membrane potential and membrane permeability. At the same time, if ROS levels accumulate to significantly high levels, it serves as a signal to initiate apoptosis, which can ultimately trigger a series of mitochondria-associated events. Our results revealed that stimulation with 2-methoxystypandrone resulted in a decrease in mitochondrial membrane potential from 97.3% to 5.15% and a significant increase in mitochondrial ROS from 1.91% to 32.7% (Figs. 3 and 4). These results therefore indicate that 2-methoxystypandrone causes abnormal oxidative metabolism, maintains the balance of ROS, and leads to oxidative stress conditions in Jurkat cells.

Antioxidant enzymes play an important role in protecting the cell molecule from free radical-generated oxidative stress. Examples of such antioxidant enzymes include HO-1, CAT, GPx, and SOD [47, 48]. HO-1 protects cells and reduces the cell-damaging effects of oxidative stress. GPx, SOD, and CAT are important components of the human body's antioxidant enzyme defense line. The collaboration of these three enzymes can remove multiple ROS to protect the body from oxidative damage. Changes in the four aforementioned antioxidant enzymes are particularly important when studying antioxidant mechanisms. According to our qPCR results, 2-methoxystypandrone significantly downregulated the mRNA expression levels of HO-1, GPx, SOD, and CAT. Therefore, 2-methoxystypandrone could be considered as an inducer of oxidative stress (Fig. 4C). These experimental results confirmed that 2-methoxystypandrone leads to a reduction in mitochondrial membrane potential and simultaneously induces the accumulation of ROS in mitochondria by inhibiting the mRNA expression levels of several key antioxidant enzymes.

In conclusion, we reported new findings on how 2-methoxystypandrone, a natural product isolated from RJH roots, targets mitochondria to inhibit the development and progression of leukemia. Our results revealed that 2-methoxystypandrone promotes apoptosis in Jurkat cells by regulating the mitochondrial pathway, which includes ROS accumulation and decreased mitochondrial depolarization. Further research confirmed that treatment with 2-methoxystypandrone delayed cell cycle progression and increased the number of apoptotic cells. Furthermore, 2-methoxystypandrone treatment resulted in decreased expression levels of Bcl_2_ and Bcl-xl and upregulated expression of BAX. In addition, we confirmed that the apoptosis promotion mechanism of 2-methoxystypandrone is linked to the downregulation of p-NF-κB p65 and phosphorylated-IκB-α. Thus, this study contributes to the investigation and application of 2-methoxystypandrone as a potential anti-leukemia agent.

## References

[1] Freireich, EJ, Wiernik PH, Steensma DP (2014). The leukemias: a half-century of discovery. J. Clin. Oncol..

[2] Lee J-E, Thuy NTT, Lee J, Cho N, Yoo HM (2019). Platyphylloside isolated from betula platyphylla is antiproliferative and induces apoptosis in colon cancer and leukemic cells. Molecules.

[3] Greaves M (2016). Leukaemia "firsts" in cancer research and treatment. Nat. Rev. Cancer.

[4] Lee J-E, Thanh Thuy NT, Lee Y, Cho N, Yoo HM (2020). An Antiproliferative ent -kaurane diterpene isolated from the roots of mallotus japonicus induced apoptosis in leukemic cells. Nat. Prod. Commun..

[5] Guo J, Cahill MR, McKenna SL, O'Driscoll CM (2014). Biomimetic nanoparticles for siRNA delivery in the treatment of leukaemia. Biotechnol. Adv..

[6] Prakash O, Kumar A, Kumar P, Ajeet A (2013). Anticancer potential of plants and natural products: a review. Am. J. Pharmacol. Sci..

[7] Cui L, Bu W, Song J, Feng L, Xu T, Liu D (2018). Apoptosis induction by alantolactone in breast cancer MDA-MB-231 cells through reactive oxygen species-mediated mitochondrion-dependent pathway. Arch. Pharm. Res..

[8] Ye YC, Wang HJ, Yu L, Tashiro SI, Onodera, Ikejima T (2012). RIP1-mediated mitochondrial dysfunction and ROS production contributed to tumor necrosis factor alpha-induced L929 cell necroptosis and autophagy. Int. Immunopharmacol..

[9] Ly JD, Grubb DR, Lawen A (2003). The mitochondrial membrane potential (δψm) in apoptosis; an update. Apotosis.

[10] Jeong SY, Seol DW (2008). The role of mitochondria in apoptosis. BMB Rep..

[11] Zhang BB, Wang, D gang, Guo F-fen, Xuan C (2015). Mitochondrial membrane potential and reactive oxygen species in cancer stem cells. Farm. Cancer.

[12] Diehn M, Cho RW, Lobo NA, Kalisky T, Dorie MJ, Kulp AN (2009). Association of reactive oxygen species levels and radioresistance in cancer stem cells. Nature.

[13] Youn J-S, Yang J, Kim S-C, Pak J-H (2019). Complete plastome sequence of *Rumex japonicus* (Polygonaceae) in Dok-do Island, Korea. Mitochondrial DNA Part B..

[14] Elzaawely AA, Xuan TD, Tawata S (2005). Antioxidant and antibacterial activities of Rumex japonicus HOUTT. aerial parts. Biol. Pharm. Bull..

[15] Liang HX, Dai HQ, Fu HA, Dong XP, Adebayo AH, Zhang LX Phytochem. Lett..

[16] Sun Y, Lenon GB, Yang AWH (2020). Rumex japonicus Houtt.: a phytochemical, pharmacological, and pharmacokinetic review. Phyther. Res..

[17] Vasas A, Orbán-Gyapai O, Hohmann J (2015). The Genus Rumex: review of traditional uses, phytochemistry and pharmacology. J. Ethnopharmacol..

[18] Chung TW, Lee J H, Choi HJ, Park MJ, Kim EY, Han JH (2017). Anemone rivularis inhibits pyruvate dehydrogenase kinase activity and tumor growth. J. Ethnopharmacol..

[19] Luo Z, Xu X, Sho T, Zhang J, Xu W, Yao J (2019). ROS-induced autophagy regulates porcine trophectoderm cell apoptosis, proliferation, and differentiation. Am. J. Physiol. Cell Physiol..

[20] Yang Z, Pan Q, Zhang D, Chen J, Qiu Y, Chen X (2019). Silibinin restores the sensitivity of cisplatin and taxol in A2780-resistant cell and reduces drug-induced hepatotoxicity. Cancer Manag. Res..

[21] Fatfat M, Fakhoury I, Habli Z, Mismar R, Gali-Muhtasib H (2019). Thymoquinone enhances the anticancer activity of doxorubicin against adult T-cell leukemia in vitro and in vivo through ROS-dependent mechanisms. Life Sci..

[22] Chen H, Zhang Y, Zhang W, Liu H, Sun C, Zhang B (2019). Inhibition of myeloid differentiation factor 2 by baicalein protects against acute lung injury. Phytomedicine.

[23] Tabuchi H, Tajimi A, Ichihara A (2014). Phytotoxic Metabolites Isolated from *Scolecotrichum graminis* Fuckel. Biosci. Biotechnol. Biochem..

[24] Nishina A, Kubota K, Osawa T (1993). Antimicrobial components, trachrysone and 2-methoxystypandrone, in *Rumex japonicus* Houtt. J. Agric. Food Chem..

[25] GUPTA SK Ascorbic Acid - Natural Sugar Lactone Esters for Comprehensive Skin & Scalp Care. U.S. Patent Application No. 12/ 139,659.

[26] Guo S, Feng B, Zhu R, Ma J, Wang W (2011). Preparative isolation of three anthraquinones from Rumex japonicus by high-speed counter-current chromatography. Molecules.

[27] Hydbring P, Malumbres M, Sicinski P (2016). Non-canonical functions of cell cycle cyclins and cyclin-dependent kinases. Nat. Rev. Mol. Cell Biol..

[28] Otto T, Sicinski P (2017). Cell cycle proteins as promising targets in cancer therapy. Nat. Rev. Cancer.

[29] Ichim G, Tait SWG (2016). A fate worse than death: apoptosis as an oncogenic process. Nat. Rev. Cancer.

[30] Seo J, Kim MW, Bae KH, Lee SC, Song J, Lee EW (2019). The roles of ubiquitination in extrinsic cell death pathways and its implications for therapeutics. Biochem. Pharmacol..

[31] Busuttil V, Bottero V, Frelin C, Imbert V, Ricci JE, Auberger P (2002). Blocking NF-κB activation in Jurkat leukemic T cells converts the survival agent and tumor promoter PMA into an apoptotic effector. Oncogene.

[32] Espinosa L, Bigas A, Mulero MC (2014). Novel functions of chromatin-bound IκBα in oncogenic transformation. Br. J. Cancer.

[33] Yu AF, Ky B (2016). Roadmap for biomarkers of cancer therapy cardiotoxicity. Heart.

[34] Comazzi S, Aresu L, Marconato L (2015). Transformation of canine lymphoma/leukemia to more aggressive diseases: anecdotes or reality? Front. Vet. Sci..

[35] Zaidman BZ, Yassin M, Mahajna J, Wasser SP (2005). Medicinal mushroom modulators of molecular targets as cancer therapeutics. Appl. Microbiol. Biotechnol..

[36] Itoigawa M (2001). Cancer chemopreventive activity of naphthoquinones and their analogs from Avicennia plants. Cancer Lett..

[37] Widhalm JR, Rhodes D (2016). Biosynthesis and molecular actions of specialized 1,4-naphthoquinone natural products produced by horticultural plants. Hortic. Res..

[38] Abraham RT, Weiss A Jurkat T cells and development of the T-cell receptor signalling paradigm. Nat. Rev. Immunol..

[39] Thuy NTT, Lee JE, Yoo HM, Cho N (2019). Antiproliferative pterocarpans and coumestans from lespedeza bicolor. J. Nat. Prod..

[40] Kuang S, Qi C, Liu J, Sun X, Zhang Q, Sima Z (2014). 2-Methoxystypandrone inhibits signal transducer and activator of transcription 3 and nuclear factor-κB signaling by inhibiting Janus kinase 2 and IκB kinase. Cancer Sci..

[41] Alenzi FQB (2004). Links between apoptosis, proliferation and the cell cycle. Br.J. Biomed. Sci..

[42] Pietenpol JA, Stewart ZA (2002). Cell cycle checkpoint signaling: cell cycle arrest versus apoptosis. Toxicology.

[43] Radha G, Raghavan SC (2017). BCL2: a promising cancer therapeutic target. Biochim. Biophys Acta Rev. Cancer.

[44] Yousef BA, Hassan HM, Zhang L-Y, Jiang Z-Z (2018). Pristimerin exhibits in vitro and in vivo anticancer activities through inhibition of nuclear factor-кB signaling pathway in colorectal cancer cells. Phytomedicine.

[45] Liang Y, Feng G, Wu L, Zhong S, Gao X, Tong Y (2019). Caffeic acid phenethyl ester suppressed growth and metastasis of nasopharyngeal carcinoma cells by inactivating the NF-&kappa;B pathway. Drug Des. Devel. Ther..

[46] Ye XQ, Li Q, Wang GH, Sun FF, Huang GJ, Bian XW (2011). Mitochondrial and energy metabolism-related properties as novel indicators of lung cancer stem cells. Int. J. Cancer.

[47] Sun J, Wei X, Lu Y, Cui M, Li F, Lu J (2017). Glutaredoxin 1 (GRX1) inhibits oxidative stress and apoptosis of chondrocytes by regulating CREB/HO-1 in osteoarthritis. Mol. Immunol..

[48] Wang S, He G, Chen, M, Zuo T, Xu W, Liu (2017). The role of antioxidant enzymes in the ovaries. Oxid. Med. Cell. Longev.

